# Early on-treatment dynamics predicting hepatitis B e antigen seroconversion in chronic hepatitis B

**DOI:** 10.3389/fimmu.2026.1816265

**Published:** 2026-06-12

**Authors:** Kai Yu, Huaguo Shao, Qiaofei Jin, Yan Zheng, Yali Hu, Shourong Liu, Xiaojing Zhang

**Affiliations:** 1 Department of Hepatology, Hangzhou Xixi Hospital Affiliated to Zhejiang Chinese Medical University (Hangzhou Sixth People’s Hospital), Hangzhou, Zhejiang, China; 2 Xixi Hospital Biobank (Clinical Data Resource Center), Hangzhou Xixi Hospital Affiliated to Zhejiang Chinese Medical University (Hangzhou Sixth People’s Hospital), Hangzhou, Zhejiang, China; 3 Department of Surgery, Hangzhou Xixi Hospital Affiliated to Zhejiang Chinese Medical University (Hangzhou Sixth People’s Hospital), Hangzhou, Zhejiang, China

**Keywords:** chronic hepatitis B, HBeAg seroconversion, LASSO logistic regression model, nucleos(t)ide analogues therapy, prediction model

## Abstract

**Background:**

Hepatitis B e antigen (HBeAg) loss with anti-HBe appearance marks the first durable immunological milestone during nucleos(t)ide analogues (NAs) therapy, yet static baseline or single time-point biomarkers anticipate this event poorly. We hypothesized that early dynamic parameters reflecting viral, antigenic and hepatic function during the first 12 weeks of therapy contain richer prognostic information than baseline values alone.

**Aims:**

This study aimed to construct a model that fuses week-12 changes in serum biochemical and virological parameters and tested its performance in predicting week-60 HBeAg seroconversion (SR).

**Methods:**

Serum biochemical and virological parameters of HBeAg-positive patients receiving NAs treatment between January and December 2023 were collected. Difference, ratio and absolute week-12 values of variables were derived. After ten-fold cross-validation, a multi-variable signature was selected to build a least absolute shrinkage and selection operator (LASSO) logistic regression model.

**Results:**

Overall, 38.24% (26/68) of patients achieved HBeAg SR. Using LASSO regression with ten-fold cross-validation, an eight-variable signature related to the dynamic changes of HBeAg, hepatitis B surface antigen (HBsAg), gamma-glutamyl transferase (GGT), and albumin (ALB) were selected. In the training cohort (n=68), the multi- variable model demonstrated an area under the curve (AUC) of 0.975 (sensitivity, 96.2%; specificity, 95.2%), significantly outperforming single-variable models. In the test cohort (n=34), the model achieved an AUC of 0.721 with maintained high specificity (95%) and positive predictive value (PPV, 90%).

**Conclusions:**

Early on-treatment integrated dynamics of HBeAg, HBsAg, GGT and ALB showed predictive value for subsequent HBeAg SR. The eight-variable model may assist monitoring or alternative strategies in patients receiving treatment.

## Introduction

Chronic hepatitis B (CHB) virus infection remains a substantial global health challenge, with an estimated 296 million people living with the disease and nearly 820,000 deaths annually from cirrhosis and hepatocellular carcinoma (1, 2). Hepatitis B e antigen (HBeAg) seroconversion (SR), defined as the loss of HBeAg with concurrent appearance of anti-HBe antibodies, represents the first major milestone of successful therapy (3). This event signals a transition from active immune-tolerant or immune-active disease to a more quiescent phase, and it correlates strongly with durable viral suppression, reduced risk of hepatic decompensation, and improved long-term survival (4, 5). Current international guidelines therefore endorse HBeAg SR as a key therapeutic endpoint, particularly for patients initiating nucleos(t)ide analogues (NAs) therapy (6, 7).

Yet the clinical reality is different. Despite potent viral suppression with first-line agents such as entecavir or tenofovir, only a few of HBeAg-positive patients achieve SR within the first year of treatment (8). The majority remain in prolonged HBeAg-positive status, facing uncertain treatment duration, accumulating drug costs, and persistent low-level oncogenic risk (9, 10). Physicians currently lack reliable tools to distinguish eventual SR at an early stage. Baseline characteristics such as alanine aminotransferase (ALT) elevation, hepatitis B virus (HBV) DNA level, or even quantitative hepatitis B surface antigen (HBsAg) provide modest discriminatory power at best, with reported area under the curve (AUC) values typically hovering around 0.7 in contemporary cohorts (11). This predictive gap forces an approach to monitoring and treatment continuation, even though the underlying biology clearly differs between patient trajectories.

It is important to acknowledge that with the widespread use of NAs, the ultimate goal of CHB treatment has shifted towards functional cure, defined as HBsAg loss. Nevertheless, HBeAg seroconversion remains a clinically relevant milestone for several reasons. First, early HBeAg seroconversion reduces the risk of HBV transmission, particularly from mother to child, which remains a major public health concern in endemic regions. Second, among HBeAg-positive patients, delayed or absent HBeAg seroconversion during NAs monotherapy often indicates suboptimal immune control, and such patients may benefit from add-on pegylated interferon therapy. Third, although HBeAg seroconversion is no longer the sole endpoint, it continues to inform clinical decision-making regarding treatment de-escalation and monitoring intervals. Therefore, an early predictive tool that identifies patients unlikely to achieve HBeAg seroconversion by week 60 could help clinicians individualize therapy, such as adding interferon or intensifying monitoring, rather than passively continuing NAs monotherapy.

The static value of pre-treatment markers evidently misses the dynamic processes that unfold during the first weeks of therapy. Viral decay, antigenic clearance, and hepatic enzyme normalization reflect the interplay of drug potency, host immune reactivation, and covalently closed circular DNA, transcriptional activity (12). These early changes, if captured quantitatively, might encode far richer prognostic information than single baseline value (13–15). Recent exploratory studies show this possibility, as demonstrated by a study assessing the predictive capacity of serum HBV RNA and HBcrAg dynamics for HBeAg SR in treatment-naïve HBeAg-positive CHB children undergoing combined therapy, which identified these markers as independent predictors and established specific decline thresholds for clinical guidance (16). However, no guideline has systematically integrated multiple parameters across viral, antigenic, and hepatic dimensions.

We therefore conducted this analysis with three specific objectives. First, we sought to derive a prediction model from early on-treatment dynamics. Second, we aimed to test this model in an independent historical cohort from the same center. Third, we compared the multi-variable model against single-variable models. Our purpose is to provide clinicians with an actionable, early prognostic tool that could inform treatment intensity and monitoring frequency before the 60-week assessment point.

## Methods

### Patients

**Figure 1:**
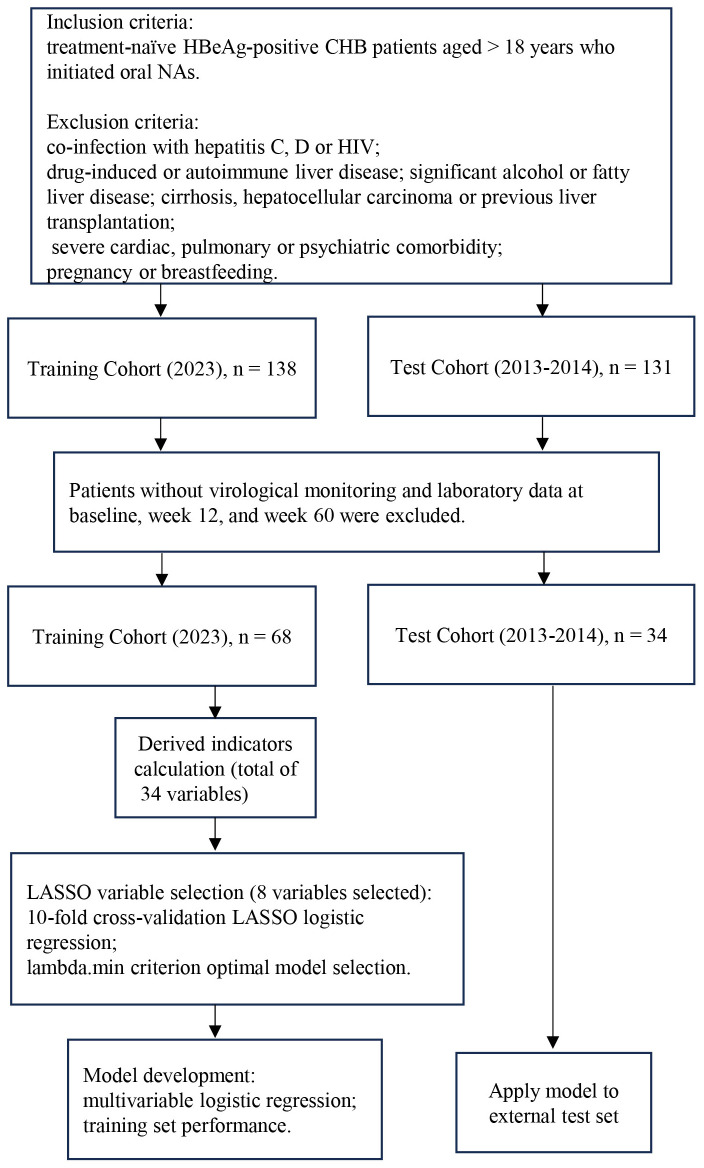
Flow diagram.

The training cohort included in this study was derived from an observational study conducted January 2023 to December 2023 (**Figure 1**). Inclusion criteria were: Treatment-naïve HBeAg-positive CHB patients aged > 18 years who initiated oral NAs, including entecavir, tenofovir disoproxil fumarate or tenofovir alafenamide were screened (17). Exclusion criteria were: co-infection with hepatitis C, D or HIV; drug-induced or autoimmune liver disease; significant alcohol (> 40 g/day) or fatty liver disease; cirrhosis, hepatocellular carcinoma or previous liver transplantation; estimated glomerular filtration rate < 60 ml/min; severe cardiac, pulmonary or psychiatric comorbidity; pregnancy or breastfeeding. Patients attended baseline and weeks 12 and 60. The actual follow-up time points for individual patients were defined as any visit occurring within ±2 weeks of the designated time points. HBeAg SR was defined as loss of HBeAg plus appearance of anti-HBe. Subjects were classified as SR or non-seroconversion (NSR) according to week-60 status. Data for the test cohort was obtained from 2013 to 2014, applying the same inclusion and exclusion criteria as the training cohort. The test cohort and the training cohort were temporally separated, with no patient overlapping or shared medical records between the two time periods.

### Laboratory assays

HBsAg were quantified by Alinity i fully automated chemiluminescent immunoassay with lower limit of 0.05 IU/ml (Abbott, USA). The assay type for HBeAg was semi-quantitative Chemiluminescence Immunoassay. Serum HBV DNA levels were measured using real-time fluorescent quantitative polymerase chain reaction (PCR) on the ABI7500 fluorescent quantitative PCR machine, with a lower detection limit of 30 IU/ml. Serum HBV RNA was measured using The HBV Ribonucleic Acid Quantitative Detection Kit of Sansure Biotech Co., Ltd. (Hunan, China). HBV RNA levels below 100 copies/ml were considered negative. Liver biochemistry was performed on Beckman Coulter AU5831. The upper limits of normal (ULN) for liver function parameters were defined as follows: ALT 50 U/L, aspartate aminotransferase (AST) 40 U/L, gamma-glutamyl transferase (GGT) 60 U/L, alkaline phosphatase 120 U/L, total bilirubin (TBIL) 23 µmol/L, and albumin (ALB) 55 g/L.

### Statistical analysis

Continuous variables were log10-transformed when distributions were right-skewed. Baseline characteristics were summarized as median, inter-quartile and range or percentage. Between-group comparisons used Wilcoxon rank-sum or χ² tests. For each analyte four parameters were calculated: (1) delta = baseline value − week-12 value; (2) ratio = week-12/baseline; (3) normalization = 1 if week-12 value ≤ ULN (≥ ULN for ALB), otherwise 0; (4) absolute week-12 value. Least absolute shrinkage and selection operator (LASSO) logistic regression with ten-fold cross-validation selected predictors of SR from 36 candidate variables. All 36 candidate variables were entered directly into the LASSO logistic regression without prior correlation pruning or univariable screening. The L1 regularization penalty inherent to LASSO shrinks coefficients of irrelevant predictors to zero, thereby performing automated variable selection. The optimal tuning parameter λ was selected using 10−fold cross−validation, and variables with non−zero coefficients at the optimal λ were retained for the final multivariable model. Multivariable logistic regression estimated coefficients and 95% confidence intervals. The optimal classification threshold for each model was determined using the Youden index, defined as the cutoff that maximizes. Model performance was assessed by area under receiver operating characteristic (ROC) curve (AUC), sensitivity, specificity, positive predictive value (PPV), negative predictive value (NPV) and accuracy. All analyses were performed in R 4.4.2 and two-sided *P* < 0.05 was considered significant.

## Results

### Development of the multi-variable model

A total of 68 patients were included in the training cohort, comprising 42 NSR and 26 SR (**Table 1**). Overall, the study population was predominantly male (68%) with a median age of 36 years. Significant differences were observed between NSR and SR groups regarding HBeAg, ALT, AST and GGT levels.

**Table 1:** Baseline clinical characteristic of training set.

Characteristic	Overall (n=68)	NSR group (n=42)	SR group (n=26)	*P* value
Age (years)	36.00(32.00,41.00)	36.00(32.00,41.00)	35.50(32.00,40.00)	0.8
Gender				0.826
Female	22 (32%)	14 (33%)	8 (31%)	
Male	46 (68%)	28 (67%)	18 (69%)	
HBsAg* (lg IU/ml)	3.88(3.55,4.23)	3.94(3.53,4.30)	3.85(3.70,4.23)	0.772
HBeAg* (lg IU/ml)	2.60(1.84,3.04)	2.85(1.94,3.06)	2.21(0.93,2.90)	0.043
HBV DNA* (lg IU/ml)	7.39(6.47,7.95)	7.44(6.22,7.95)	7.28(6.48,7.87)	0.825
HBV RNA* (lg copies/ml)	7.19(6.75,7.70)	7.19(6.73,7.70)	7.23(6.97,7.70)	0.923
ALT (U/L)	246.00(80.50,673.00)	194.00(61.00,477.00)	358.50(200.00,914.00)	0.015
AST (U/L)	137.00(63.50,337.00)	101.00(53.00,255.00)	169.00(103.00,374.00)	0.048
TBIL (U/L)	19.26(11.92,36.35)	18.36(11.47,30.19)	25.27(15.16,41.04)	0.331
ALB (U/L)	38.00(35.50,41.15)	38.20(34.90,43.00)	37.65(35.70,40.20)	0.965
ALP (U/L)	93.50(74.00,122.00)	90.50(73.00,125.00)	101.00(75.00,116.00)	0.905
GGT (U/L)	78.00(35.00,162.50)	52.50(33.00,106.00)	154.50(51.00,235.00)	0.013

*HBsAg, HBeAg, HBV DNA and HBV RNA levels were log10-transformed.

**Figure 2:**
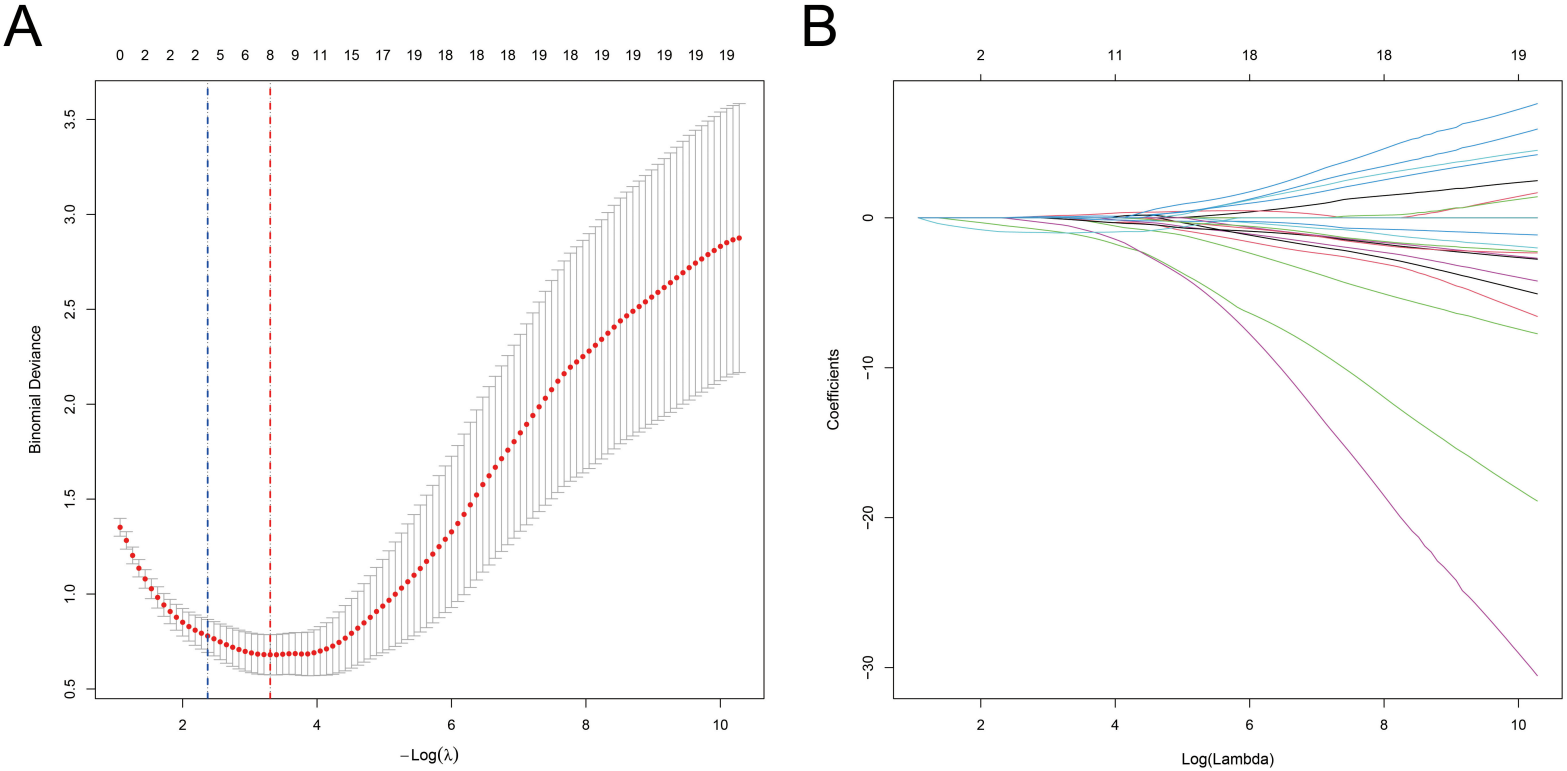
Feature selection using LASSO logistic regression. **(A)** Ten-fold cross-validation for tuning parameter (λ) selection. The left dashed vertical line indicates the optimal λ value corresponding to the minimum mean cross-validated deviance, while the right dashed line represents the largest λ within one standard error of the minimum. **(B)** Coefficient path profiles of the candidate predictors. Each colored line represents the trajectory of an individual variable’s regression coefficient as the regularization parameter log(λ) changes.

To comprehensively capture the treatment response dynamics, we generated four categories of candidate predictors from baseline and week 12 data, resulting in a total of 36 candidate variables entering the subsequent selection process. Using 10-fold cross-validation with the training cohort (n=68), we evaluated the binomial deviance across a range of regularization parameters (λ). As shown in **Figure 2A**, the cross-validated error reached its minimum at λ.min, corresponding to a model containing 8 non-zero coefficients. **Figure 2B** displays the coefficient paths across the regularization spectrum. As λ decreased, predictors entered the model sequentially based on their discriminative importance. The final selected variables comprised “delta HBsAg”, “delta HBeAg”, “ratio HBeAg”, “ratio ALB”, “ratio GGT”, “week 12 HBsAg”, “week 12 HBeAg” and “week 12 GGT”, each contributing independently to the prediction of sustained response.

Multivariable logistic regression analysis confirmed the independent contributions of the eight selected variables (**Supplementary Table 1**). Among these, ratio HBeAg (OR = 0.05; 95% CI: 0.00-0.48; *P* = 0.035), ratio GGT (OR = 0.46; 95% CI: 0.00-0.56; *P* = 0.011), week 12 GGT (OR = 0.96; *P* = 0.036), and ratio ALB (OR = 0.47; 95% CI: 0.00-14.8; *P* = 0.021) were significant negative predictors of HBeAg SR. The intercept term was also significant (OR = 25.8; 95% CI: 1.42-46.8; *P* = 0.045). Delta HBsAg (OR = 1.35; *P* = 0.073), week 12 HBsAg (OR = 0.50; *P* = 0.052) and week 12 HBeAg (OR = 2.64; *P* = 0.707) were not statistically significant.

We subsequently constructed a multivariate logistic regression model incorporating these selected variables. In the training cohort, this multi-variable model demonstrated discriminative ability with an AUC of 0.975, significantly outperforming any single-variable model (**Table 2**). The model achieved optimal calibration with 96.2% sensitivity, 95.2% specificity, and 95.6% overall accuracy, indicating robust classification of treatment responders.

**Table 2:** Performance of all models in the training cohort.

Indicator	AUC	Sensitivity	Specificity	Accuracy	PPV	NPV
LASSO eight-variable	0.975	0.962	0.952	0.956	0.926	0.976
HBeAg (ratio)	0.942	0.115	0.119	0.118	0.075	0.179
HBeAg (week 12)	0.924	0.269	0	0.103	0.143	0
HBeAg (delta)	0.85	0.846	0.714	0.765	0.647	0.882
GGT (ratio)	0.754	0.346	0.214	0.265	0.214	0.346
HBV RNA (ratio)	0.743	0.231	0.31	0.279	0.171	0.394
GGT (delta)	0.728	0.808	0.571	0.662	0.538	0.828
HBV RNA (delta)	0.717	0.808	0.667	0.721	0.6	0.848
ALT (ratio)	0.702	0.385	0.238	0.294	0.238	0.385
HBV RNA (week 12)	0.702	0.5	0.143	0.279	0.265	0.316
AST (ratio)	0.698	0.154	0.452	0.338	0.148	0.463
HBsAg (ratio)	0.691	0.115	0.476	0.338	0.12	0.465
HBsAg (delta)	0.68	0.885	0.5	0.647	0.523	0.875
AST (delta)	0.679	0.885	0.524	0.662	0.535	0.88
ALT (delta)	0.677	0.885	0.452	0.618	0.5	0.864
ALP (week 12)	0.654	0.5	0.19	0.309	0.277	0.381
HBsAg (week 12)	0.644	0.269	0.452	0.382	0.233	0.5
GGT (week 12)	0.644	0.308	0.429	0.382	0.25	0.5
AST (week 12)	0.643	0.115	0.595	0.412	0.15	0.521
AST (normalization)	0.642	0.808	0.476	0.603	0.488	0.8
HBV DNA (ratio)	0.636	0.154	0.571	0.412	0.182	0.522
ALT (week 12)	0.634	0.615	0.119	0.309	0.302	0.333
HBV DNA (delta)	0.62	0.692	0.571	0.618	0.5	0.75
HBV DNA (week 12)	0.618	0.077	0.667	0.441	0.125	0.538
ALP (ratio)	0.611	0.654	0.048	0.279	0.298	0.182
ALP (delta)	0.596	0.462	0.762	0.647	0.545	0.696
TBIL (week 12)	0.561	0.5	0.69	0.618	0.5	0.69
ALT (normalization)	0.554	0.846	0.262	0.485	0.415	0.733
ALB (delta)	0.55	0.385	0.452	0.426	0.303	0.543
TBIL (delta)	0.548	0.808	0.357	0.529	0.438	0.75
ALB (ratio)	0.548	0.654	0.524	0.574	0.459	0.71
ALB (week 12)	0.542	0.192	0.952	0.662	0.714	0.656
GGT (normalization)	0.502	0.885	0.119	0.412	0.383	0.625
ALP (normalization)	0.502	0.885	0.119	0.412	0.383	0.625
TBIL (ratio)	0.453	0.538	0.548	0.544	0.424	0.657
ALB (normalization)	0.5	0	1	0.618	NaN	0.618
TBIL (normalization)	0.5	0	1	0.618	NaN	0.618

NaN: Not a number.

We further assessed the stability and overfitting of the LASSO model using bootstrap internal validation with 1000 resamples. As shown in **Supplementary Figure 1**, the original AUC in the training cohort was 0.975, and the mean bias−corrected AUC was 0.858, with the 95% confidence interval indicated by the shaded area. The gap between the original and corrected AUC reflects optimism due to the limited sample size, indicating a certain degree of overfitting. Nevertheless, the corrected AUC remained as high as 0.858, suggesting that the model retains good inherent discriminative ability.

### Comparison between multi-variable model and single-variable models

**Figure 3:**
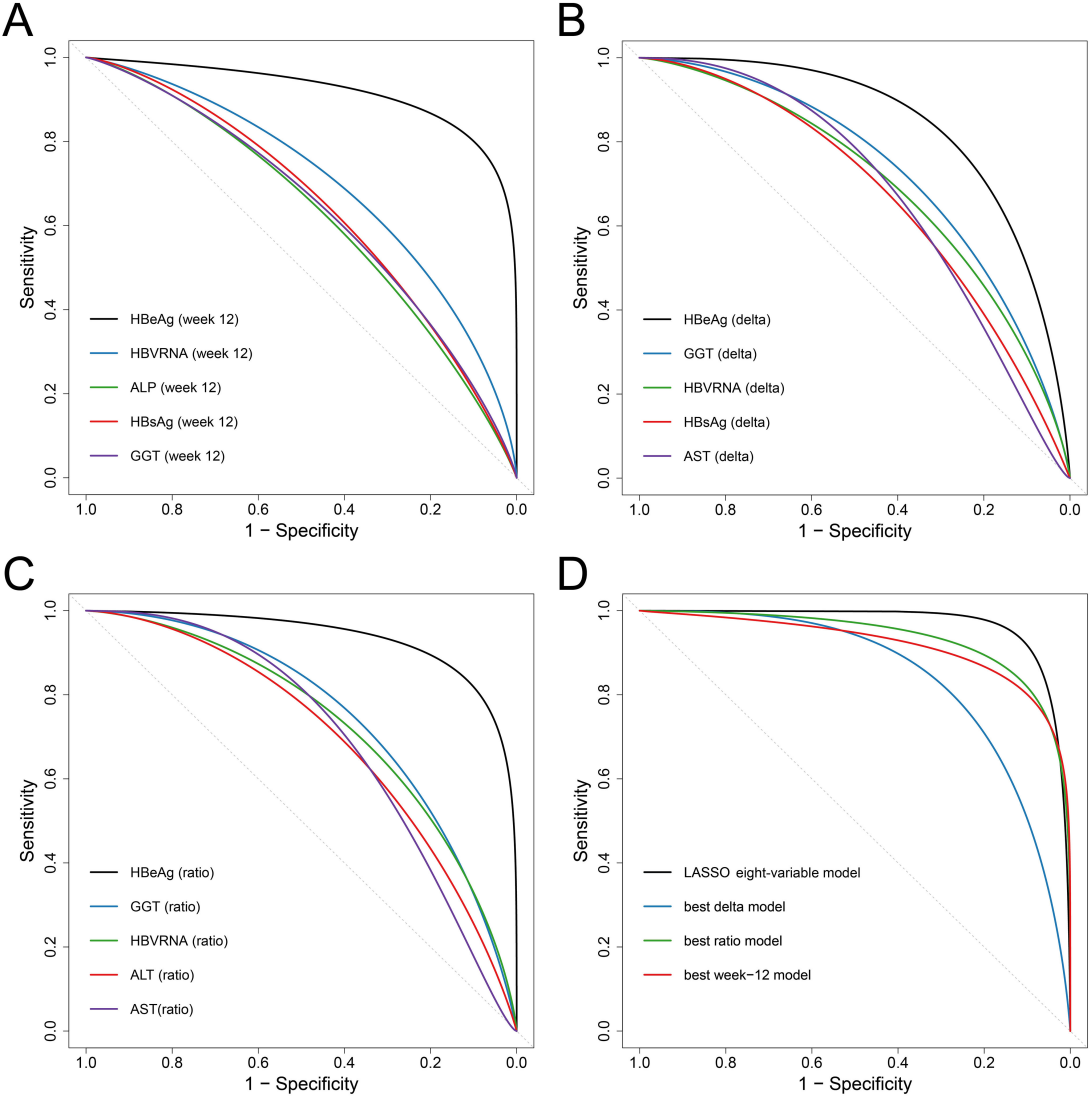
Predictive performance in the training cohort assessed by ROC curve analysis. ROC curves for **(A)** absolute values at week 12, **(B)** delta values, **(C)** ratio values of candidate biomarkers. **(D)** Comparative ROC curves of the LASSO multi-variable model (black line) against the best-performing single-parameter models selected from each category. The diagonal dotted line represents the reference line of no discrimination (AUC = 0.5).

The LASSO multi-variable model demonstrated superior discriminative performance compared to all individual biomarkers. As shown in **Table 2**, the multi-variable model achieved an AUC of 0.975, significantly higher than other single-variable model, ratio HBeAg model (AUC = 0.942), week 12 HBeAg model (AUC = 0.924) and delta HBeAg model (AUC = 0.850), while most biochemical parameters showed only modest predictive accuracy (**Figures 3A–C**).

Beyond AUC values, the multi-variable model exhibited an optimal balance between sensitivity and specificity, achieving 96.2% sensitivity and 95.2% specificity with an overall accuracy of 95.6%. In contrast, although ratio HBeAg model possessed the highest AUC among individual markers, its clinical utility was severely limited by extremely low sensitivity (11.5%) and specificity (11.9%), leading to poor predictive values (PPV, 7.5%; NPV, 17.9%). Similarly, week 12 HBeAg model showed inadequate sensitivity (26.9%) despite its high AUC. Among the single-variable models, only delta HBeAg model provided relatively balanced sensitivity (84.6%) and specificity (71.4%), yet its accuracy (76.5%) remained inferior to the multi-variable model.

**Figure 3D** visually illustrates that the ROC curve of the LASSO multi-variable model consistently dominated the curves of the best-performing single-variable models across all threshold settings. These results indicated that integrating multiple complementary biomarkers effectively overcomes the inherent limitations of univariate predictors, thereby enhancing both the accuracy and robustness of the prediction.

### Performance in the test cohort

**Figure 4:**
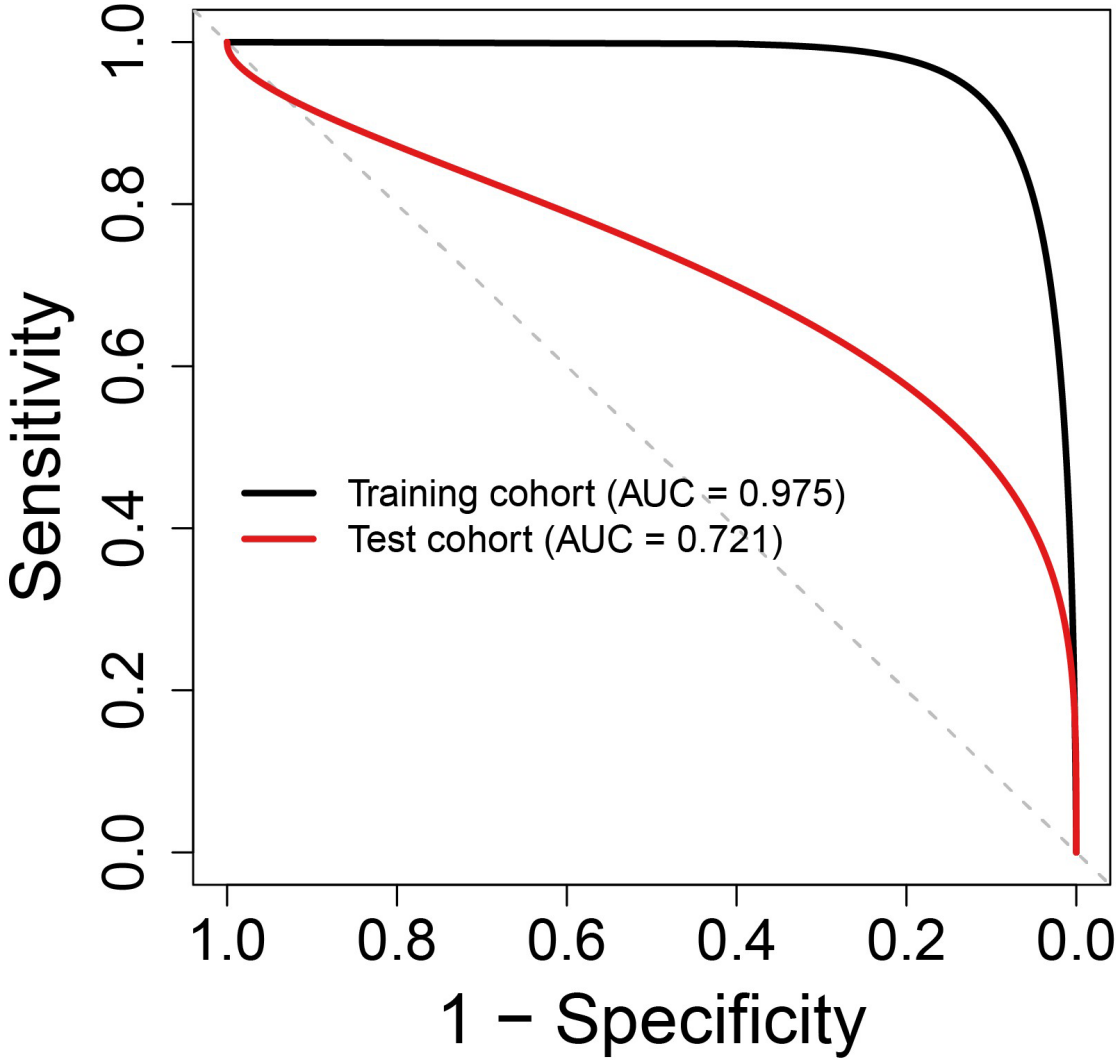
ROC curves of the eight-variable LASSO logistic regression model in the training and test cohorts. The model achieved an AUC of 0.975 in the training cohort (n=68) and an AUC of 0.721 in the test cohort (n=34). The diagonal dotted line represents the reference line of no discrimination (AUC = 0.5).

The performance of the model was evaluated on test cohort comprising 34 patients. The clinical characteristics of the test cohort are presented in **Table 3**. Of these, 14 were assigned to the SR group and 20 to the NSR group. In the test cohort, the model achieved an AUC of 0.721, with a sensitivity of 0.643, a specificity of 95%, an overall accuracy of 82.35%, a PPV of 90% and NPV of 79.17%, respectively (**Figure 4**). in the test cohort, while the model maintained high specificity for NSR (95.0%, 19/20), its sensitivity for SR decreased substantially to 64.3% (9/14), yielding five false-negative predictions. Consequently, the overall accuracy in the test cohort was 82.4% (28/34). The model exhibited a conservative prediction tendency in the test cohort, with only one false-positive but five false-negatives, resulting in a PPV of 90.0% and NPV of 79.2%.

**Table 3:** Baseline clinical characteristic of test set.

Characteristic	Overall (n=34)	NSR group (n=20)	SR group (n=14)	*P* value
Gender				0.076
Female	11 (32%)	9 (45%)	2 (14%)	
Male	23 (68%)	11 (55%)	12 (86%)	
HBsAg* (lg IU/ml)	3.11(2.44,3.31)	3.19(2.95,3.36)	2.42(2.12,3.18)	0.013
HBeAg* (lg IU/ml)	0.53(0.29,0.95)	0.61(0.35,0.87)	0.41(0.15,0.97)	0.162
HBV DNA* (lg IU/ml)				0.76
<3	23 (68%)	14 (70%)	9 (64%)	
≥3	6 (18%)	4 (20%)	2 (14%)	
Missing	5 (14%)	2 (10%)	3 (22%)	
ALT (U/L)	30.00(25.00,40.00)	30.00(24.00,44.00)	31.00(25.00,40.00)	0.7
AST (U/L)	31.00(24.00,41.00)	31.00(24.50,40.50)	32.00(24.00,42.00)	0.82
TBIL (U/L)	13.35(10.60,17.30)	11.75(9.65,14.15)	17.05(12.80,18.70)	0.011
ALB (U/L)	45.40(44.60,46.90)	45.80(44.85,47.30)	44.90(44.00,46.50)	0.221
ALP (U/L)	90.50(74.00,115.00)	84.00(69.50,114.00)	104.00(79.00,115.00)	0.172
GGT (U/L)	37.00(26.00,65.00)	29.50(23.50,49.00)	40.00(31.00,133.00)	0.103

*HBsAg, HBeAg and HBV DNA levels were log10-transformed.

## Discussion

We integrated early on-treatment changes in HBeAg, HBsAg, GGT and ALB to build a discriminative model for HBeAg SR. The eight-variable signature predicted week-60 HBeAg SR status with 95.6% accuracy in the training cohort and 82.35% in the test cohort, outperforming each single biomarker.

The 95.2% and 95% specificity of training cohort and test cohort enable reliable identification of patients unlikely to seroconvert by week 60. Such individuals could be prioritized for add-on peg-interferon, short-interval monitoring or counselling on long-term therapy. Conversely, those with high predicted probability might safely extend visit intervals, reducing healthcare burden. Integration of the model into electronic health records could automate risk calculation and facilitate shared decision-making.

Previous studies have explored diverse predictive strategies for HBeAg seroconversion (SR), evolving from static baseline characteristics to dynamic monitoring paradigms. Traditional static markers, such as baseline HBsAg and HBV DNA, have shown limited discriminatory capacity, with reported AUC values typically between 0.60 and 0.73 (18). Recent investigations have therefore focused on dynamic single-variable assessments. Serum HBV RNA kinetics at week 6 or 24 have emerged as robust predictors, with Liu et al. reporting an AUC of 0.942 for week-24 HBV RNA levels (19). Similarly, Hong et al. demonstrated that the trajectory of HBeAg decline over 12 months, combined with age at treatment initiation, predicted functional cure in children (AUC 0.83) (20). Beyond conventional serology, epigenetic markers such as PEG10 and TUBB1 promoter methylation have shown independent predictive value, with AUCs of 0.895 and 0.805, respectively (21, 22). Nevertheless, these approaches have notable limitations: most rely on a single biomarker, some require late time points, and others depend on specialized technical platforms, limiting their routine clinical applicability. Our study directly addresses these gaps. First, methodologically, we integrate four biological dimensions viral antigens, cholestasis, and hepatic synthetic functions into a single model, capturing the synergistic effects of viral suppression, inflammation resolution, and functional recovery. Second, mechanistically, the inclusion of GGT and ALB reveals that successful SR requires not only viral suppression but also concurrent improvement in liver function. GGT is a marker of cholestasis and oxidative stress, both of which are closely linked to hepatic inflammation and immune dysregulation (23). Elevated GGT reflects active biliary damage and the production of reactive oxygen species, which can impair antigen presentation and T−cell function. An early decline in GGT during treatment may therefore signal resolution of oxidative stress and restoration of the hepatic immune microenvironment, facilitating subsequent HBeAg seroconversion. ALB, as a measure of hepatic synthetic reserve, reflects global hepatocellular function (24). Low ALB is associated with chronic inflammation and reduced immune competence (25). Improvement in ALB during the first 12 weeks of therapy may indicate restoration of normal hepatocyte metabolism and a more permissive immune environment for HBeAg seroconversion. Third, regarding application value, all our variables are derived from routine laboratory tests, avoiding specialized platforms. Our model uses week-12 dynamics, substantially earlier than the week-24 time point used in previous HBV RNA studies. Additionally, its 95% specificity reliably identifies patients unlikely to achieve SR, enabling timely clinical decisions such as add-on peginterferon or intensified monitoring. Collectively, these innovations enhance the model’s clinical translatability and distinguish our work from prior studies. The clinical utility of this model rests on two complementary rationales. First, early identification of patients unlikely to achieve HBeAg seroconversion enables timely intervention, such as add-on pegylated interferon, which has been shown to increase the seroconversion rate in NAs non-responders. Second, from a public health perspective, achieving earlier HBeAg seroconversion reduces the risk of HBV transmission, particularly in mother-to-child transmission scenarios. Thus, the model’s high specificity can reliably rule in patients who are unlikely to seroconvert, allowing clinicians to consider alternative strategies rather than continuing NAs monotherapy passively. However, it is important to note that the low sensitivity remains a limitation potentially leading to unnecessary treatment escalation if the model were used alone.

This study has several limitations. First, the training and test cohorts were derived from distinct time periods, which may introduce historical or temporal bias. Over the past decade, advances in virological assay sensitivity, laboratory platforms, and clinical management strategies for chronic hepatitis B have inevitably altered baseline patient characteristics and measurement precision. These differences could partially account for the attenuation of model performance observed in the test cohort. The AUC in the test cohort was substantially lower than that in the training cohort, primarily due to profound baseline differences in disease activity between the two cohorts (**Supplementary Table 2**). The training cohort consisted of patients with active hepatitis, whereas the test cohort comprised patients with quiescent or inactive disease. This discrepancy reflects the evolution of chronic hepatitis B treatment guidelines over the past decade. Notably, the test cohort still maintained high specificity and positive predictive value, indicating that the model retains clinical utility for identifying patients unlikely to achieve seroconversion. Importantly, however, the variables incorporated into the eight-variable signature model consist exclusively of routinely measured and clinically standardized biomarkers, including HBeAg, HBsAg, GGT, and ALB. Moreover, the model emphasizes early on-treatment dynamics, expressed as relative changes and ratios, rather than absolute baseline values, which may mitigate the impact of inter-era assay variability. In this context, test set using an earlier cohort provides a stringent test of model robustness under less standardized conditions. The reduced discriminative performance in the historical cohort, therefore, likely represents a conservative estimate of the model’s true predictive capacity rather than a fundamental limitation. Nevertheless, these findings underscore the necessity of future prospective, multi-center validation using contemporaneous cohorts and harmonized laboratory platforms before clinical implementation. Second, despite the use of LASSO regularization, the potential for model overfitting cannot be excluded. The eight-variable signature model was derived from a relatively small training cohort with a limited number of seroconversion events, which may inflate apparent performance, particularly in internal evaluation. Regarding the lower bounds of some confidence intervals appearing as “0.00”, this is a consequence of the L1 regularization in LASSO regression, which strongly shrinks coefficients of weakly associated predictors. Some variables in **Supplementary Table 1** do not reach the conventional significance threshold. This might appear contradictory to the strong overall predictive performance of the LASSO model; however, the set of variables selected by LASSO exhibits a joint effect, and the model’s predictive power derives from their synergistic contribution rather than from any single variable’s independent effect. The substantial reduction in discriminative ability observed in the test cohort underscores this concern. The eight-variable signature model was derived from a relatively small training cohort with a limited number of seroconversion events. The events-per-variable (EPV) ratio was 3.25, which falls below the minimum recommended threshold of 10 for logistic regression models. This low EPV is the root cause of model overfitting, likely to explain both the optimism observed in the bootstrap-corrected AUC and the performance attenuation in the test cohort. Accordingly, the present model should be regarded as exploratory and hypothesis-generating rather than a ready-to-use clinical prediction tool. Its primary value lies in highlighting the prognostic relevance of early on-treatment dynamics across viral, antigenic, and hepatic functional domains, rather than in providing definitive individual-level predictions. Prospective validation in larger, contemporary, multi-center cohorts is required before clinical implementation can be considered. Third, our model was developed exclusively in patients receiving NAs, its applicability to peg-interferon monotherapy or combination regimens remains unproven. Finally, the binary classification does not capture the spectrum of partial treatment responses or the timing of SR beyond week 60.

In future studies, the eight-variable signature should be validated using larger multi-center cohorts. Integration of novel immunological markers, such as HBcrAg kinetics, quantitative anti-HBc, or PD-1 expression on T cells, may further enhance predictive accuracy by capturing host immune status more comprehensively. A two-stage prediction framework combining baseline and early on-treatment biomarkers at week 4 or week 8 should be established to better characterize the trajectory toward SR. Additionally, machine learning approaches such as random forests or gradient boosting machines could be compared with LASSO regression to optimize predictive performance in larger datasets.

## Conclusion

Early on-treatment integrated dynamics of HBeAg, HBsAg, GGT and ALB showed predictive value for subsequent SR. The eight-variable signature model may assist monitoring or alternative strategies in patients receiving treatment.

## Data Availability

The original contributions presented in the study are included in the article/**Supplementary Material**. Further inquiries can be directed to the corresponding author/s.

## Supplementary material

Supplementary Figure 1 Bootstrap internal validation of the LASSO model The histogram shows the distribution of AUC values obtained from 1000 bootstrap resamples. The original AUC in the training cohort was 0.975 (indicated by the vertical line). The mean bias-corrected AUC was 0.858, representing a more robust estimate of the model’s expected performance on internal validation. The shaded area indicates a 95% confidence interval.
