# Supplementary figures and images for: Early on-treatment dynamics predicting hepatitis B e antigen seroconversion in chronic hepatitis B
Source: Front Immunol. 2026 Jun 12;17:1816265. doi: 10.3389/fimmu.2026.1816265 (PMC13303963; doi:10.3389/fimmu.2026.1816265)

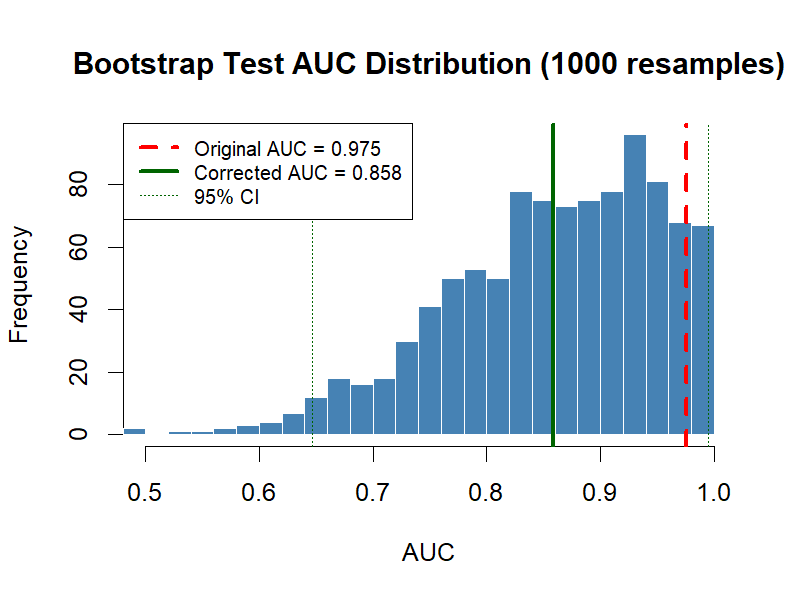

Supplement: Supplementary Figure 1 — Bootstrap internal validation of the LASSO model The histogram shows the distribution of AUC values obtained from 1000 bootstrap resamples. The original AUC in the training cohort was 0.975 (indicated by the vertical line). The mean bias-corrected AUC was 0.858, representing a more robust estimate of the model’s expected performance on internal validation. The shaded area indicates a 95% confidence interval. [file Image1.png]
