# Supplementary material for: Early on-treatment dynamics predicting hepatitis B e antigen seroconversion in chronic hepatitis B
Source: Front Immunol. 2026 Jun 12;17:1816265. doi: 10.3389/fimmu.2026.1816265 (PMC13303963; doi:10.3389/fimmu.2026.1816265)
Supplement: Supplementary file 2 [file Table1.docx]

Supplementary Table 1. Logistic regression table.

| Indicator | OR | 95% CI | *P* value |
| --- | --- | --- | --- |
| (Intercept) | 25.8 | 1.42, 46.8 | 0.045 |
| Delta HBsAg | 1.35 | 0.24, 9.25 | 0.073 |
| Delta HBeAg | 0.69 | 0.03, 6.04 | 0.079 |
| Ratio HBeAg | 0.05 | 0.00, 0.48 | 0.035 |
| Ratio GGT | 0.46 | 0.00, 0.56 | 0.011 |
| Ratio ALB | 0.47 | 0.00, 14.8 | 0.021 |
| Week 12 HBsAg | 0.50 | 0.05, 4.44 | 0.052 |
| Week 12 HBeAg | 2.64 | 0.08, 160 | 0.707 |
| Week 12 GGT | 0.96 | 0.88, 1.02 | 0.036 |
