# Supplementary material for: Early on-treatment dynamics predicting hepatitis B e antigen seroconversion in chronic hepatitis B
Source: Front Immunol. 2026 Jun 12;17:1816265. doi: 10.3389/fimmu.2026.1816265 (PMC13303963; doi:10.3389/fimmu.2026.1816265)
Supplement: Supplementary file 3 [file Table2.docx]

Supplementary Table 2. Comparison of baseline characteristics between the training cohort and test cohort.

| Characteristic | Training cohort (n = 68) | Test cohort (n = 34) | *P* value |
| --- | --- | --- | --- |
| Gender |  |  | 1 |
| Female | 22 (32%) | 11 (32%) |  |
| Male | 46 (68%) | 23 (68%) |  |
| HBsAg* (lg IU/ml) | 3.88 (3.55, 4.23) | 3.11(2.44,3.31) | <0.001 |
| HBeAg* (lg IU/ml) | 2.60 (1.84, 3.04) | 0.53(0.29,0.95) | <0.001 |
| ALT (U/L) | 246.00 (80.50,673.00) | 30.00 (25.00,40.00) | <0.001 |
| AST (U/L) | 137.00 (63.50,337.00) | 31.00 (24.00,41.00) | <0.001 |
| TBIL (U/L) | 19.26 (11.92,36.35) | 13.35 (10.60,17.30) | 0.002 |
| ALB (U/L) | 38.00 (35.50,41.15) | 45.40 (44.60,46.90) | <0.001 |
| ALP (U/L) | 93.50 (74.00,122.00) | 90.50 (74.00,115.00) | 0.622 |
| GGT (U/L) | 78.00 (35.00,162.50) | 37.00 (26.00,65.00) | 0.001 |

*HBsAg and HBeAg levels were log10-transformed.
